# Telemonitoring and Quality of Life in Patients after 12 Months Following a Pacemaker Implant: the Nordland Study, a Randomised Trial

**DOI:** 10.3390/ijerph16112001

**Published:** 2019-06-05

**Authors:** Remedios López-Liria, Antonio López-Villegas, Terje Enebakk, Hilde Thunhaug, Knut Tore Lappegård, Daniel Catalán-Matamoros

**Affiliations:** 1 Department of Nursing, Physiotherapy and Medicine, Health Research Centre, University of Almeria, 04120 Almeria, Spain; 2 Social Involvement of Critical and Emergency Medicine, CTS–609 Research Group, Poniente Hospital, 04700 Almería, Spain; 3 Division of Medicine, Nordland Hospital, N-8092 Bodø, Norway; Terje.Enebakk@nordlandssykehuset.no (T.E.); Hilde.Thunhaug@nordlandssykehuset.no (H.T.); knut.tore.lappegard@gmail.com (K.T.L.); 4 Institute of Clinical Medicine, Faculty of Health Sciences, University of Tromsø, N-9037 Tromsø, Norway; 5 Department of Journalism and Communication, Universidad Carlos III de Madrid, 28903 Madrid, Spain; dcatalan@ual.es; 6 Health Sciences CTS–451 Research Group, University of Almeria, 04120 Almeria, Spain

**Keywords:** long term, pacemaker follow-up, quality of life, remote monitoring, telemedicine

## Abstract

The purpose of this study was to analyse the health-related quality of life (HRQoL) of patients followed up using a remote device-monitoring system (TM) compared to patients followed up through standard outpatient visits (HM), 12 months after the implantation of a pacemaker. This was a trial design that used the EuroQol-5D Questionnaire and the Minnesota Living with Heart Failure Questionnaire (MLHF). The HRQoL of a cohort of 50 consecutive patients randomly allocated to one of the two follow-up modalities was measured at baseline and then during follow-up, 12 months after the pacemaker implantation. Eventually, 23 patients were followed-up through standard outpatient visits, while 23 used a remote monitoring system. Results: The baseline clinical characteristics and health-related quality of life of the patients from both groups were observed to be statistically similar. Twelve months after the pacemaker implantation, both groups showed statistically significant improvements in the baseline parameters based on the MLHF. The patients followed up through hospital visits showed a greater increase in MLHF-HRQoL after 12 months, although the increase was not significantly greater than that of the TM group. Furthermore, the frequencies of emergency visits and re-hospitalisations did not differ between the groups.

## 1. Introduction

The number of patients with chronically implanted cardiovascular implantable electronic devices (CIEDs) is continuously increasing, and device clinics at hospitals may soon be unable to fully cope with the increasing number of follow-up activities [1]. Current guidelines necessitate one or two follow-up assessments per year for patients implanted with a pacemaker (PM) [2].

The World Health Organisation (2010) has defined telemedicine as “the delivery of healthcare services by all healthcare professionals using information and communication technologies for the exchange of valid information for the diagnosis, treatment, and prevention of disease and injuries, where distance is a critical factor” [3]. It offers an opportunity for delivery of specialist services closer to patients’ homes. Research has demonstrated that telemedicine can significantly reduce both rates of mortality and re-hospitalisation in patients implanted with PM. Furthermore, it allows a medical practitioner to communicate remotely with them, thus saving money on both transportation and time. Consequently, healthcare costs have been considerably reduced with the use of these technologies [4,5,6,7].

The objective of disease management is to reduce operating costs without lowering effectiveness by creating disease-specific pathways of diagnosis and care in patients at a high risk of heart failure, thus allowing doctors to quickly detect and respond to adverse events, which may also improve patients’ health status [1,8]. Previous studies have reported that although telemedicine requires an initial financial investment, such interventions eventually result in a substantial reduction in the medical costs over a long period of time [9].

Currently, the outcomes of patients with cardiac implantable electronic devices, including improved quality of life of patients, reduced need for in-office interrogation, patient safety, and enhanced survival—which remains the most significant benefit—demonstrate the effectiveness of remote monitoring [10]. In patients implanted with pacemakers (PM), telemedicine potentially facilitates patient safety through early detection of arrhythmia or device malfunction, allowing earlier intervention, which ultimately improves the patients’ survival rate [1,11].

In order to improve outcomes such as the health-related quality of life (HRQoL) of patients implanted with PM, various multidisciplinary projects have been developed over the previous years across various countries in Europe [12,13,14]. Hole et al. (2010) demonstrated that reduced quality of life as assessed by the Minnesota Living with Heart Failure Questionnaire (MLHF) score among elderly heart failure (HF) patients in Norway is a strong predictor of death. Improving the quality of daily life of these patients should be a primary goal, along with improving symptoms and prognosis [15]. This method of disease monitoring may allow specialist services to increase the number of patients under their care [16].

Due to an insufficient number of comparable randomised trials assessing HRQoL in patients using a telemonitoring (TM) system in the long term [7], it remains unclear whether TM approaches provide extra benefits in terms of the daily life of patients with heart failure, one year after hospitalisation [17] in a setting where geographical effects could possibly influence the results. Therefore, we hypothesised that patients receiving a remote device-monitoring follow-up system would have lower post-discharge resource use (re-admission rates, time to first re-admission, duration of hospital stays, and urgent care-clinic visits), higher 12-month survival rates, and improved quality of life compared to patients receiving standard ambulatory-based follow-up after discharge. The purpose of this study was to analyse the health-related quality of life perceived by patients with telemonitoring of pacemakers (TM), compared to patients followed up through standard outpatient visits (HM: Hospital monitoring), 12 months after PM implantation.

## 2. Materials and Methods

### 2.1. Design and Participants

This controlled, randomised, non-masked clinical trial enrolled 50 patients implanted with a single or dual-chamber pacemaker (PM) at Nordland Hospital (Bodø, Norway). Although the trial protocol was previously described in detail [18], it is necessary to explain that the study enrolment commenced in August 2014. All patients were included consecutively in the study and were followed up for 12 months after pacemaker implantation, until October 2016. All of them fulfilled the inclusion criteria (age > 18 years, ability to provide informed consent and operate the home monitor, life expectancy > one year) and none of the exclusion criteria (scheduled for implantable cardioverter-defibrillator (ICD) or cardiac resynchronisation therapy (CRT) and participation in other trials). We used data collected from the NORDLAND patient cohort for this analysis (Supplementary Materials) [18]. All of the participants were recruited from the Nordland Hospital and the investigators had access to the electronic charts covering every contact with the hospital during follow-up [18]. Twenty-five patients were allocated to the telemonitoring programme (Group TM) and the other 25 (Group HM) were followed up using the standard protocol. Because of the explicit nature of the intervention, it was not possible to blind patients or staff to the identity of the group to which they had been randomised with sealed envelopes.

**Figure 1:**
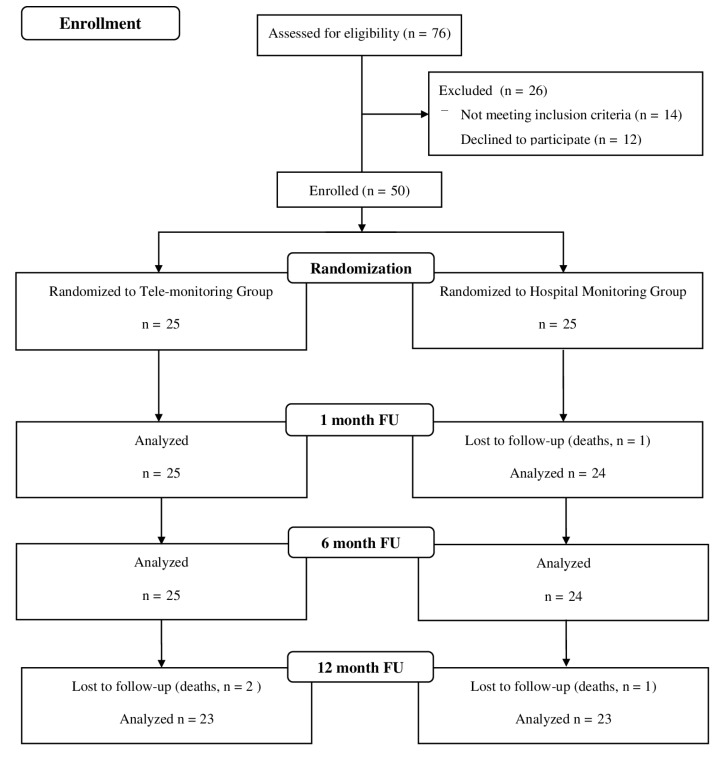
Flow (CONSORT) diagram of the study.

The questionnaires on HRQoL were personally administered to patients included in both groups on the date of pacemaker implantation during hospitalisation and via telephone at one, six, and 12 months after discharge. Figure 1 illustrates the enrolment process.

### 2.2. Ethics Approval and Consent for Participation

The protocol was approved by the Regional Health Research Ethical Committee (REK Nord, Tromsø, Norway, with the reference number 2014/383/REK Nord). The study was developed in accordance with the principles of the Declaration of Helsinki. The trial protocol was registered in ClinicalTrials.gov (Identifier: NCT02237404). All patients signed the corresponding written informed consent prior to their enrolment, and appropriate measures were taken to ensure data privacy.

### 2.3. Instruments

Socio-demographic data and baseline characteristics of the study sample were described, and two sets of the questionnaire, EuroQol-5D (EQ–5D) [19] and MLHF [20], were used to evaluate the quality of life.

The EQ-5D is a generic questionnaire about HRQoL, which is simple and easy to complete, which makes it attractive for use [21,22]. It is composed of two different sections: The first section involves a patient-provided subjective evaluation of five aspects (mobility, self-care, daily activities, pain/discomfort, and anxiety/depression state), and the second section includes a visual-analogue scale from 0 (the worst health state) to 100 (the best health state). Cronbach’s co-efficient (α) was 0.73, indicating that the instrument was reliable [23].

The MLHF was used owing to its widespread use, comparability, and a large number of available publications [20,24]. Knox, Rahman & Beedie (2017) reported that the MLHF was the most commonly used measure [7]. This questionnaire consists of 21 questions regarding patients’ perception of the effect of heart failure on their daily lives, wherein the score for each question ranges from 0 to 5. Based on the answers, a total score of 0 to 105 is obtained, with a higher score indicating a poorer quality of life. Rector et al. (1987) suggested that the MLHF is a valid self-assessment of the therapeutic benefit of medical therapy by the patient (Cronbach’s α for all items was 0.92) [20].

Other measures of outcome were the differences between the TM and HM group with regard to 1) follow-up visits or in-clinic visits, 2) cardiovascular adverse events, 3) changes of medication and PM reprogramming, and 4) hospitalisation after implant, duration, and reasons for hospitalisation.

All patients underwent an in-clinic follow-up six weeks after the implantation of a pacemaker. Patients allocated to the group receiving routine follow-ups were then scheduled for another visit 12 months after the implantation, whereas the TM group was summoned to the hospital when alerted by the system. If referred by their general practitioner, patients in both groups were seen in the hospital according to routine local guidelines.

### 2.4. Devices

Based on their diagnosis, patients received either a single (VVIR) or a dual-chamber (DDDR) pacemaker. Patients in the TM group received either a Biotronik Estella SR-T/DR-T or a Biotronik Evia SR-T/DR-T (Biotronik SE&Co KG, Berlin, Germany), whereas patients of the HM group received either of the aforementioned Biotronik pacemakers, a St Jude Medical Endurity SR/DR (St Jude Medical, Sylmar, CA, USA), or a Sorin Reply 200 SR/DR (LivaNova PLC, London, United Kingdom).

Home monitoring was performed through the Biotronik Home Monitoring^®^ system (Biotronik SE&Co KG, Berlin, Germany)—an internet-based TM service for users with Biotronik implantable heart devices. Biotronik devices equipped with Home Monitoring (T-devices) have additional storage capacity and contain a small RF-antenna for wireless communication and data transmission from the implant to a wireless patient device, the CardioMessenger (Biotronik SE&Co KG, Berlin, Germany). Home Monitoring has no negative effects on the battery life of the device (Telecardiology system BIOTRONIK).

Every night, the CardioMessenger automatically collects and transmits important encrypted health information to the Biotronik service centre using the global network of T-Mobile and its partners (GPRS). The transmitted patient data are collected, automatically analysed, and segregated at the Biotronik Home Monitoring Service Centre, depending on the patient needs defined by their physician. Health- and system-related issues are ranked and marked in order of importance. All event and trend reports can be accessed and reviewed on a protected online platform. Furthermore, according to pre-set definitions, the physician may receive automatic warnings (e.g., by e-mail or text message) concerning safety issues including premature battery depletion and lead fracture.

The alerts generated by the telemonitoring were managed by the implanting clinic. There were automatic routine transfers every three months and the clinic was notified by an e-mail from the Home Messenger system. If the e-mail read "No events" no further action was taken. In the case of alerts the investigating physician would contact the patient, and depending on the nature of the alert the patient would be summoned to a check-up at the hospital or changes in medication would be made (e.g., anticoagulation or increased anti-arrhythmic treatment in the case of atrial fibrillation).

### 2.5. Statistical Analysis

The NORDLAND study design and data analysis method are described in detail elsewhere [18]. The groups were compared using a difference in the means test for continuous variables and a difference in the proportions test (binomial method) or Chi-Square test (replaced by the Fisher´s exact test for more than five cases) for qualitative variables. Inter-group differences in the pre-specified endpoints were also assessed using the difference in means or proportion tests and Wilcoxon-signed ranks test for the MLHF questionnaire. The results including the corresponding 95% confidence intervals (95% CI) were presented. All analyses were carried out using the standard statistical software (SPSS Version 23.0, SPSS Inc., Chicago, Illinois, USA).

## 3. Results

The mean age was 75 ± 12 years, and 48% of the patients were females. The most common pacing indication was sick sinus syndrome (48%), followed by atrioventricular block (40%), and chronic atrial fibrillation (AF) with bradycardia (12%). Dizziness was the most common symptom (50%), and the patients were most commonly referred from the hospital´s in-house cardiology ward or from other local hospitals. In 88% of the cases, dual-chamber pacemakers were used. Hypertension was the most frequent comorbidity (64%), followed by dyslipidaemia (54%) and tachyarrhythmia (36%) [18].

The baseline clinical characteristics including HRQoL of the participants enrolled in our study were uniform and without statistically significant differences between the two groups (Table 1 and Table 2).

In the baseline analysis, the patients had mean scores of 0.78 (95% CI: 0.72; 0.85) for EQ-5D utilities and 64.83 (95% CI: 56.74; 70.14) for EQ-5D VAS. Twelve months after PM implantation, the scores varied to 0.76 (95% CI: 0.71; 0.88) and 70.09 (95% CI: 64.36; 75.81), respectively (Table 1). The changes between baseline and 12-month EQ-5D utilities and VAS were not statistically significant (*p* = 0.53 and *p* = 0.65, respectively). No significant differences were observed in these parameters or any of the EQ-5D utility dimensions between the TM and HM group at enrolment and 12 months (Table 3).

Regarding HRQoL results at 12 months, the EQ-5D utility score of all patients (Table 3) had decreased 0.02 points lower than the baseline and the mean of EQ-5D VAS was 5.26 points higher. Although the HRQoL scores with EQ-5D improved over time across both groups, there were no statistically significant differences between initial and final assessment. 

At the baseline assessment, the HM group had a higher MLHF score (28.96) (poorer quality of life) than the TM group (20.20), although the difference was not statistically significant. At 12 months also, there were no significant differences between the two groups (TM: 9.17; HM: 10.78. *p* = 0.45) (Table 2). The MLHF scores (Table 3) demonstrated a significant improvement in both groups (−14.17 points; 95% CI: 8.24; 20.10) compared to the baseline scores (*p* < 0.001). At the end of the study period, the HM group experienced a better response compared to the baseline (TM: –10.67; HM: –16.57).

During the 12-month period of the follow-up, 6% of the patients had experienced at least one adverse cardiovascular event (AE) (TM: 8%, HM: 4%; *p* = 0.39) (Table 4), and 26% of the patients had been hospitalised at least once (TM: 28%, HM: 32%; *p* = 0.53). The main reasons for hospitalisation were coronary problems (TM: 16%; HM: 24%) and PM dysfunction (TM: 12%; HM: 0%); however, no significant differences were observed between the groups. Four patients (TM: 2; HM: 2) died from non-cardiovascular causes. The findings indicated no differences in the time to re-hospitalisations or emergency visits between the TM and HM group. There were no differences in the duration of hospital stay or use of urgent care clinic. The number of in-clinic visits due to “changes of pacemaker reprogramming” was 11 in the TM group, and in the HM group it was 6; this difference was not statistically significant (*p* = 0.311).

## 4. Discussion

The aim of our study was to evaluate the relevant clinical values and the HRQoL in a random sample of PM users of a TM system at the 12-month follow-up and compare them with the baseline data at discharge from the hospital. There is a lack of such randomised trials in patients using a TM system in the long term, to which data from standard monitoring systems could be compared [7,17]. The present study has shown that pacemaker-implanted patients followed up via a TM system yielded similar outcomes as those followed up with standard outpatient visits. TM was well received and accepted by the patients.

Similar results were reported by Ricci et al. (2017) who assessed the costs and benefits of TM compared to the conventional follow-up from the TARIFF patient cohort (prospective, non-randomised, multicentre clinical trial) [25]. The EuroQoL EQ-5D-3L questionnaire was implemented for each patient at discharge and at the 12-month follow-up. It was found that the quality-adjusted life-years of the patients did not significantly differ between the groups, irrespective of the scenario. These findings are similar to those observed in our study using the EQ-5D questionnaire. In addition, the TM of users with cardiac implantable electronic devices was reported to have reduced costs with regard to the healthcare system, patients, and caregivers [25].

The BEAT-HF study [17] was one of the largest clinical trials on telemonitoring of HF patients, six months after implantation of PM. Among the 1437 randomised participants using either TM or HM, no significant differences were observed in 30-day readmission or 180-day mortality, but the 180-day HRQoL differed significantly between the two groups, i.e. individuals participating in TM may experience improvements at 180 days, using the MLHF. In the Nordland study, the difference observed in the MLHF score between the groups could be because the patients receiving conventional monitoring at the hospital started the study with a worse quality of life and ended up with a quality of life similar to the TM group. There was a difference between baseline and 12-month scores that could also be due to the fact that health-related quality of life was measured using MLHF, which is specific for heart failure (higher sensitivity in assessing HRQoL in PM patients) compared to the more generalised EQ-5D Questionnaire. Indeed, EQ-5D was not able to detect differences in the quality of life between the TM and HM group of patients, which was also demonstrated in previous research [26].

In the Comoretto study (2016) on a cohort of 42 consecutive patients, the PM-specific Assessment of Quality of Life and Related Events Questionnaire indicated that the use of a TM system increased the HRQoL of PM patients after three months following device implantation, compared to those with standard follow-up [26]. However, in a study on long-term telemonitoring of PM patients (the COMPAS randomised, multicentre, non-inferiority trial) [12], a comparative follow-up schedule with a home monitoring trial showed that the differences in HRQoL between the remotely monitored patients and those followed up using standard methods were not statistically significant. The results of the study are similar to the findings of the Nordland study. In another study, although TM did not have a significantly greater impact on the mental and physical quality of life (QoL) compared to the usual care, it was associated with a small but significant increase in overall QoL [7].

Dar et al. (2009) examined the impact of home TM on typical HF patients discharged from three hospitals in North West London (there were no significant differences between the groups) [16]. They reported similar findings to our study at 12 months of follow-up and found no change in overall health-related quality of life assessed via the EQ-5D over a six-month follow-up period. However, QoL measured through the disease-specific MLHF improved slightly. The authors suggested that TM in a typical elderly population of HF patients not only produced a similar outcome to “usual” specialist care but also reduced clinic and emergency room visits and unplanned HF re-hospitalisations at little additional cost. The PONIENTE study (a controlled, non-randomised, non-blinded clinical trial) proposed that the remote monitoring of pacemakers in older adults was an equivalent option to hospital follow-up with regard to HRQoL and functional capacity after 12 months [14].

Our study showed a similarity in the time to re-hospitalisation or emergency visits between the TM and HM group of patients, differing from a study by Antonicelli et al. (2008) on 57 congestive HF patients randomised into standard care and home telemonitoring-based care; after following these patients for 12 months, the authors reported that TM was associated with improvements in the composite endpoint of mortality and rate of hospitalisation, better compliance to therapy, and an improved reported health perception score (SF–36), possibly due to the better compliance and closer monitoring of the patients [27]. The remote monitoring of HF patients differs from our study in the way that several HF devices are capable of detecting fluid retention at an early stage and can report such a deterioration to the responsible clinician. This can, in turn, result in an increase in the dose of diuretics and thus prevent hospitalisation.

A Cochrane review and a meta-analysis concluded that telemonitoring reduces all-cause mortality and HF-related hospitalisations [5,28]. Another systematic review reported that telemonitoring systems helped in the early detection of cardiovascular events compared to conventional monitoring, thus reducing the number of hospitalisations and hospital visits and lowering the costs associated with follow-ups [29].

It has been established in the recently published HRS (Heart Rhythm Society) consensus document that TM tenders a magnificent opportunity to enhance the efficiency of clinics and enable continuous monitoring of the functioning of the device and clinical status of patients and supported adherence to disease management programmes [9,30]. TM has been associated with high patient acceptance, satisfaction, and increased adherence to scheduled follow-up, providing many advantages to patients and clinicians [31,32,33].

Nordland County has a scattered population of 6.3 inhabitants/sq km. Although a large proportion of the population in Nordland County live in cities, these are small, and there remains a significant fraction of inhabitants living in communities with fewer than 2,500 individuals at great distances from the hospital. Previous publications indicate that the healthcare needs of patients living in rural areas usually involve having problems with access to healthcare [34,35]. Telemonitoring of pacemakers offers an opportunity for the delivery of specialist services closer to patients’ homes where distance is a critical factor [18].

The results obtained in our study should be considered under the following limitations: 1) Due to the small sample size, it could be possible that a larger randomised trial might have possibly detected significant statistical differences between the two groups, and the results could differ in different Health Centre Systems and social settings. Further investigations should focus more on the randomised design of trials with large and homogenous participants in the two groups. 2) The positive effect of telemonitoring observed in this study may in part be due to the fact that the most positive or healthiest patients were in the telemonitoring group (random allocation), in contrast to several previous studies. 3) Depending on the diagnosis, patients included in the TM group could receive two different pacemakers from the same company, and users included in the conventional monitoring group (HM) could be implanted with four different types of pacemakers (from three different companies). In the basal evaluation, however, after the HRQoL questionnaire assessment, no significant differences were found between the two groups. On the other hand, we only evaluated the automatic wireless RM (Remote monitoring) technology from one company. Thus, our results may not apply to other proprietary technologies with varying transmission frequencies and methods of alert notification.

From our point of view, further studies are needed to confirm these findings as, to the best of our knowledge, more evidence is required due to the limited number of studies. Apart from the therapeutic and diagnostic benefits of PM, improvement in clinical outcomes, clinical efficiencies, and patient experience could be assessed for implantable cardioverter-defibrillators, cardiac resynchronisation therapy devices, and implantable loop recorders, with the adoption of remote CIED monitoring. These benefits confer significant value to both CIED follow-up centres and patients [36].

The TM technology, introduced in Europe for the management of CIEDs in the 21st century, has spread rapidly and become a standard mode of medical care for patients where distance is a critical factor. A TM system could reduce the consumption of healthcare resources (saving money on transportation and time), including hospitalisations for cardiovascular reasons, emergency department visits, outpatient diagnostic tests, outpatient clinical evaluation visits, and device follow-up visits. This preference for remote monitoring could perhaps be extended to countries with geographic dispersion, or to patients hampered by mobility problems, work schedules, or poor traffic conditions, once demonstrated that the quality of life of these patients is not affected negatively with respect to other patients whose follow-up is performed by the physician in the hospital.

Telemonitoring users have also witnessed important technological changes such as the increasing utilisation of tablet computers and other dispositive or remote sensors. Recent approaches such as implantable devices could facilitate adherence or provide better information in the detection of problems after discharge [17].

## 5. Conclusions

This study used a randomised trial to demonstrate that the HRQoL of users with pacemakers improved in both the TM and HM group after PM implantation. MLHF questionnaire scores showed a higher increase of HRQoL in users monitored at the hospital. However, the quality of life assessed using the EQ-5D questionnaire did not differ significantly between the two groups at any stage. In this study, the MLHF questionnaire appeared to measure quality of life more accurately than the EQ-5D one.

The results presented in this study indicate that telemonitoring is an equally effective alternative to the conventional follow-up in the hospitals and provides similar outcomes regarding clinic and emergency room visits and unplanned re-hospitalisations.

## Figures and Tables

**Table 1:** Health-related quality of life at 12 months of follow-up.

EQ-5D Utilities		Month 0				Month 12			
		TM n (%)	HM n (%)	Total	p	TM n (%)	HM n (%)	Total	p
MOBILITY	No problems	15 (60.0)	12 (48.0)	27 (54.0)	0.29	16 (69.6)	11 (47.8)	27 (58.7)	0.12
	Some problems	10 (40.0)	13 (52.0)	23 (46.0)		7 (30.4)	12 (52.2)	19 (41.3)	
	Extreme problems	0 (0.0)	0 (0.0)	0 (0.0)		0 (0.0)	0 (0.0)	0 (0.0)	
SELF-CARE	No problems	22 (88.0)	22 (88.0)	44 (88.0)	0.67	23 (100.0)	22 (95.7)	45 (97.8)	0.50
	Some problems	22 (88.0)	22 (88.0)	6 (12.0)		0 (0.0)	1 (4.3)	1 (2.2)	
	Extreme problems	0 (0.0)	0 (0.0)	0 (0.0)		0 (0.0)	0 (0.0)	0 (0.0)	
USUAL ACTIVITIES	No problems	16 (64.0)	14 (56.0)	30 (60.0)	0.84	14 (60.9)	13 (56.5)	27 (58.7)	0.50
	Some problems	8 (32.0)	10 (40.0)	18 (36.0)		9 (9.1)	10 (43.5)	19 (41.3)	
	Extreme problems	1 (4.0)	1 (4.0)	2 (4.0)		0 (0.0)	0 (0.0)	0 (0.0)	
PAIN/DISCOMFORT	No problems	11 (44.0)	14 (56.0)	25(50.0)	0.08	9 (39.1)	12 (52.2)	21 (45.7)	0.48
	Some problems	14 (56.0)	8 (32.0)	22 (44.0)		11 (47.8)	10 (43.5)	21 (45.7)	
	Extreme problems	0 (0.0)	3 (12.0)	3 (6.0)		3 (13.0)	1 (4.3)	4 (8.7)	
ANXIETY/DEPRESSION	No problems	16 (64.0)	16 (64.0)	32 (64.0)	0.62	20 (87.0)	21 (91.3)	41 (89.1)	0.50
	Some problems	9 (36.0)	9 (36.0)	18 (36.0)		3 (13.0)	2 (8.7)	5 (10.9)	
	Extreme problems	0 (0.0)	0 (0.0)	0 (0.0)		0 (0.0)	0 (0.0)	0 (0.0)	
EQ-5D Utilities	TOTAL (95CI)	0.81 (0.74;0.87)	0.76 (0.64;0.88)	0.78 (0.72;0.85)	0.47	0.73 (0.68;0.91)	0.78 (0.74;0.91)	0.76 (0.71;0.88)	0.53
EQ-5D VAS	TOTAL (95CI)	64.00 (55.77;72.23)	64.88 (51.69;74.07)	64.83 (56.74;70.14)	0.86	71.52 (63.47;79.57)	68.65 (59.91;77.40)	70.09 (64.36;75.81)	0.65

TM: Telemonitoring group; HM: Hospital monitoring group; N (month 0) = 50 (TM group: 25; HM group: 25). N (month 1) = 49 (TM group: 25; HM group: 24). N (month 6) = 49 (TM group: 25; HM group: 24). Values are expressed as means (95CI: 95% confidence interval of means). EQ-5D: EuroQoL-5D; VAS: Visual Analog Scale.

**Table 2:** Minnesota Living with Heart Failure questionnaire (MLHF) scores.

MLHF Items	Month 0			Month 12		
	TM *n* = 25	HM *n* = 25	*p*	TM *n* = 23	HM *n* = 23	*p*
Swelling in ankles, legs	0.84	1.00	0.62	0.29	0.71	0.25
Resting during the day	1.28	2.08	0.22	0.81	1.48	0.09
Walking and climbing stairs	0.96	1.92	0.28	0.38	0.48	0.79
Working around the house	1.20	2.36	0.33	0.52	0.95	0.31
Going away from home	0.28	1.44	0.90	0.62	0.24	0.29
Sleeping	0.84	1.32	0.37	0.38	0.43	0.89
Doing things with friends or family	0.88	1.96	0.70	0.62	0.86	0.61
Working to earn a living	0.40	0.72	0.73	0.05	0.05	1.00
Recreational pastimes	1.24	1.76	0.08	0.38	0.95	0.16
Sexual activities	0.32	0.68	0.95	0.14	0.19	0.83
Eating less of the food liked	0.60	1.00	0.21	0.29	0.29	1.00
Shortness of breath	2.44	2.48	0.87	1.10	1.76	0.22
Fatigue, tiredness, low on energy	2.36	3.20	0.78	0.86	1.57	0.19
Staying in the hospital	1.48	1.76	0.95	0.14	0.00	0.33
Costing money for medical care	0.60	0.80	0.21	0.19	0.10	0.61
Side effects from medication	0.44	0.68	0.67	0.43	0.05	0.07
Feeling burdensome	0.32	0.76	0.57	0.00	0.00	1.00
Feeling a loss of self-control	0.80	0.76	0.22	0.19	0.48	0.36
Worrying	1.00	0.88	0.83	0.38	0.24	0.63
Difficulty remembering & concentrating	1.16	0.96	0.70	0.48	0.33	0.67
Feeling depressed	0.44	0.76	0.96	0.29	0.19	0.72
TOTAL (95%CI)	20.20 (14.48; 25.92)	28.96 (19.97;37.95)	0.07	9.17 (4.05;14.29)	10.78 (5.52;16.05)	0.45

TM: Telemonitoring group; HM: Hospital monitoring group; N (month 0) = 50 (TM group: 25; HM group: 25).

N (month 1) = 49 (TM group: 25; HM group: 24). N (month 6) = 49 (TM group: 25; HM group: 24).

Item values are expressed as means. Total values are expressed as means (95CI: 95% confidence interval of means).

**Table 3:** Differences in health-related quality of life at 12 months of follow-up.

Questionnaires	All				Telemonitoring				Hospital Monitoring			
**Health-Related Quality of Life–Specific**												
	Baseline	Month 12	Differences	*p*	Baseline	Month 12	Differences	*p*	Baseline	Month 12	Differences	*p*
MLHF (95CI)	24.58 (19.30;29.86)	9.96 (6.42;13.49)	14.17 (8.24; 20.10)	<0.001	20.20 (14.48;25.92)	9.17 (4.05;14.29)	10.67 (4.98;16.36)	0.001	28.96 (19.97–37.95)	10.78 (5.52–16.05)	16.57 (7.54;25.60)	0.001
**Health-Related Quality of Life–General**												
EQ5D VAS (95CI)	64.83 (56.74;70.14)	70.09 (64.36;75.81)	5.26 (2.44;12.96)	0.18	64.00 (55.77;72.23)	71.52 (63.47;79.57)	6.52 (3.16;16.20)	0.18	64.88 (51.69;74.07)	68.65 (59.91;77.40)	3.77 (0.45;8.26)	0.66
EQ-5D utilities (95CI)	0.78 (0.72;0.85)	0.76 (0.71;0.88)	0.02 (−0.11;0.07)	0.62	0.81 (0.74;0.87)	0.73 (0.68;0.91)	0.08 (−0.12;0.10)	0.84	0.76 (0.64;0.88)	0.78 (0.74;0.91)	0.02 (−0.12;0.07)	0.53

N (month 0) = 50 (TM group: 25; HM group: 25). N (month 12) = 46 (TM group: 23; HM group: 23). Values are expressed as means (95CI: 95% confidence interval of means). EQ-5D: EuroQoL-5D; VAS: Visual Analog Scale; MLHF: Minnesota Living with Heart Failure questionnaire.

**Table 4:** Follow-up information at 12 months.

Variables	*n* (%)	TM *n* = 23	HM *n* = 23	*p*-Value
**In-clinic visits**				
0	1 (2.0)	0 (0.0)	1 (4.0)	0.297
1	41 (82.0)	21 (84.0%)	20 (80.0)	
2	6 (12.0)	2 (8.0)	4 (16.0)	
3	2 (4.0)	2 (8.0)	0 (.0)	
**Transmissions from patients’ home**				
0	30 (60.0)	5 (20.0)	25 (100.0)	<0.001
3–5	15 (30.0)	15 (60.0)	0 (0.0)	
6–8	5 (10.0)	5 (20.0)	0 (0.0)	
**Cardiovascular adverse events**				
None	47 (94.0)	23 (92.0)	24 (96.0)	0.388
PCI	1 (2.0)	1 (4.0)	0 (0.0)	
Angina	1 (2.0)	0 (0.0)	1 (4.0)	
Lead dislodgement x 2	1 (2.0)	1 (4.0)	0 (0.0)	
**Calls/letters sent to the patients**				
0	28 (56.0)	4 (16.0)	24 (96.0)	<0.001
1	21 (42.0)	20 (80.0)	1 (4.0)	
3	1 (2.0)	1 (4.0)	0 (0.0)	
**Changes of medication**				
0	34 (68.0)	17 (68.0)	17 (68.0)	0.107
1	7 (14.0)	5 (20.0)	2 (8.0)	
2	3 (6.0)	1 (4.0)	2 (8.0)	
3	4 (8.0)	0 (0.0)	4 (16.0)	
4	2 (4.0)	2 (8.0)	0 (0.0)	
**Changes of PM reprogramming**				
0	35 (70.0)	16 (64.0)	19 (76.0)	0.311
1	13 (26.0)	7 (28.0)	6 (24.0)	
2	2 (4.0)	2 (8.0)	0 (0.0)	
**Hospitalisation number after implant**				
0	30 (60.0)	14 (56.0)	16 (64.0)	0.532
1	15 (30.0)	7 (28.0)	8 (32.0)	
2	4 (8.0)	3 (12.0)	1 (4.0)	
5	1 (2.0)	1 (4.0)	0 (0.0)	
**Days spent hospitalised**				
0	30 (60.0)	14 (56.0)	16 (64.0)	0.535
1–5	12 (24.0)	6 (24.0)	6 (24.0)	
6–10	5 (10.0)	2 (8.0)	3 (12.0)	
+11	3 (6.0)	3 (12.0)	0 (0.0)	
**Reasons for hospitalisation**				
None	30 (60.0)	14 (56.0)	16 (64.0)	0.333
Other	6 (12.0)	3 (12.0)	3 (12.0)	
Cancer	1 (2.0)	1 (4.0)	0 (0.0)	
Coronary problems	10 (20.0)	4 (16.0)	6 (24.0)	
PM disfunction	3 (6.0)	3 (12.0)	0 (0.0)	

## Supplementary Materials

The following are available online at https://www.mdpi.com/1660-4601/16/11/2001/s1, Table S1: CONSORT 2010 checklist of information to include when reporting a randomised trial.
